# CD4^+^ Memory Stem T Cell in Peripheral Blood: A Promising Immune Index for Early Screening and Auxiliary Diagnosis of Colorectal Cancer

**DOI:** 10.3389/fonc.2021.701738

**Published:** 2021-07-13

**Authors:** Yan Lu, Qiaohong Zhang, Longyi Zhang

**Affiliations:** Clinical Laboratory, DongYang People’s Hospital, Dongyang, China

**Keywords:** memory stem T cell, colorectal cancer, auxiliary diagnosis, lymphocytes, early screening

## Abstract

**Background and Aims:**

Colorectal cancer (CRC) lacks obvious symptoms in the early stage of the disease, making it is easy to be misdiagnosed and remain undetected. Here, we explored the role of CD4^+^ memory stem T cells (TSCM) in peripheral blood in the early screening and auxiliary diagnosis of CRC.

**Materials and Methods:**

Patients diagnosed with a “colorectal mass” by colonoscopy, at the Dongyang People’s Hospital (Zhejiang, China), between November 2020 and June 2021, were included in this prospective study. Using histopathological results as the gold standard for diagnosis, patients were divided into “CRC group” and “benign tumor group”. Healthy volunteers were recruited as “healthy controls.” Ten-color flow cytometry was used to detect CD4^+^ T cell subsets, and the results were analyzed using the Kaluza software. Carcinoembryonic antigen (CEA) and carbohydrate antigen 199 (CA199) were detected by the Roche Cobas e 602 electrochemiluminescence immunoassay analyzer.

**Results:**

This study involved 33 patients with CRC, 41 patients with colorectal benign tumors, and 49 healthy volunteers. The absolute value and frequency of CD4^+^ TSCM can clearly distinguish colorectal cancer, benign tumors, and healthy controls. According to the area under the receiver operating characteristic curve (AUC), the absolute value of CD4^+^ TSCM used to assist in the diagnosis of CRC was 0.758 (sensitivity: 0.612; specificity: 0.788), which is higher than the values for CEA (AUC: 0.707) and CA199 (AUC: 0.552). In early screening, the sensitivity of the absolute value of CD4^+^ TSCM (sensitivity: 0.612) was significantly higher than that of CEA (sensitivity: 0.333) and CA199 (sensitivity: 0.259).

**Conclusion:**

CD4^+^ TSCM in peripheral blood may be a promising immune index for the early screening and auxiliary diagnosis of CRC.

## Introduction

Colorectal cancer (CRC) is the fourth most common type of cancer in China (1). In 2020, China was estimated to have the largest number of CRC cases worldwide, and the incidence and mortality of CRC has increased in recent decades (2, 3). CRC is prone to misdiagnosis and missed diagnosis due to the lack of obvious symptoms in the early stage of the disease. Delay in treatment is considered an independent risk factor for poor prognosis of colorectal cancer (3). However, simply using a conventional tumor marker does not meet the needs of early screening and diagnosis (4). Therefore, exploring the peripheral blood markers for early screening and auxiliary diagnosis has become a topic of interest in CRC research. These advances will improve the early diagnosis rate and discovery of clinical treatment targets for CRC.

It is well known that immune cells have anti-tumor activity (5) and are activated in the early stage of cancer to participate in the body’s immune response. They are considered as promising diagnostic markers and therapeutic targets. However, not every type of immune cell has the prospect of a clinical application. Studies have focused on immune cells showing rapid proliferation and strong immune effects, because such cells can benefit patients with CRC (6).

T cells are the most common immune cells in the tumor microenvironment, and the immunity mediated by them is an important component of the human immune system (7). When an oncogenic pathogen invades the body, naive T cells (TNs) with multidirectional differentiation ability differentiate into functional subpopulations, including central memory T cells (TCMs) and effector memory T cells (TEMs), to achieve a rapid, durable, and effective immune response upon re-stimulation by oncogenic pathogens (8). The process of cell differentiation is continuous, with the naive cells having a better differentiation ability and the effector function of the cells getting stronger closer to the end of progression (9). Recently, memory stem T cells (TSCM) were discovered, as a new T-cell subpopulation, by Luca Gattinoni et al. (10). The differentiation stage of TSCM is between that of TN and TCM, and these cells not only have the ability of self-renewal, rapid proliferation, and differentiation like TNs but also have as strong an immune activity as TEMs (11). Klebanoff et al. (12) showed that TSCM have the strongest self-renewal capacity and anti-tumor activity among all T cell subsets in animals (13).

CD8^+^ T lymphocyte subsets have been extensively studied for their direct involvement in antitumor effects (13–15). Recent studies have found that CD4^+^ T lymphocytes play an integral role in the induction of tumor cell regression by immunotherapy (16). Here, we explored the correlation between CD4^+^ TSCM expression and CRC by analyzing peripheral blood, with the aim of providing new research directions for the early screening and auxiliary diagnosis of CRC.

## Materials and Methods

### Study Population

This prospective study included patients who visited the Dongyang People’s Hospital between November 2020 and June 2021 and were diagnosed with a “colorectal mass” *via* colonoscopy. The histopathology results of tissue specimens were used as the basis for the diagnosis of colorectal cancer. The exclusion criteria were as follows: ① the patients refused to be further diagnosed by pathology; ② CRC was not the primary lesion or was combined with other tumors; ③ missing clinicopathological information; and ④ the patient had chronic persistent human immunodeficiency virus, hepatitis C virus, or human papilloma virus infection. The study was approved by the ethics committee of the Dongyang People’s Hospital, and all participating patients signed an informed consent form.

### Sample Collection and Detection

**Figure 1:**
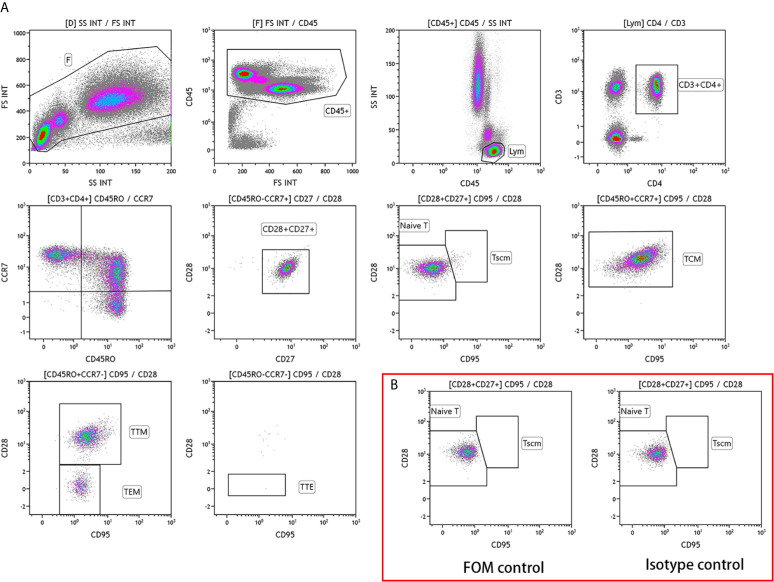
Analysis scheme of CD4^+^ T lymphocyte subsets in the peripheral blood of patients with colorectal cancer **(A)** Phenotype of CD4^+^ T lymphocyte subsets. TSCM, memory stem T cells; TN, naive T cells; TCM, central memory T cells; TTM, terminal memory T cells; TEM, effector memory T cells; TTE, terminal effector T cells. **(B)** Control scheme.

Fasting venous blood (2 mL) was collected from participants in the study and control groups before surgery, and flow cytometry was performed within 24 h. Nucleated cells were stained with the following antibodies: CD3-Alexa Fluor 750 (clone UCHT1; Beckman Coulter, USA), CD4-FITC (clone 13B8.2; Beckman Coulter), CD8-Alexa Fluor 700 (clone B9.11; Beckman Coulter), CD28-PC5.5 (clone CD28.2; Beckman Coulter), CD27-PC7 (clone 1A4CD27; Beckman Coulter), CD45-Kro (clone J.33; Beckman Coulter), CD45RO-ECD (clone UCHL1; Beckman Coulter), CD95-PE (DX2; BD Biosciences, UK), and CCR7-APC (G043H7; BioLegend). The ten-color flow cytometer (Naivos; Beckman Coulter) was used for detection. The test results were analyzed using the Kaluza software (version 2.0, Beckman Coulter). The analysis scheme is illustrated in **Figure 1**.

Carcinoembryonic antigen (CEA) and carbohydrate antigen 199 (CA199) were detected by an electrochemiluminescence immunoassay (ECLIA). The Roche Cobas e602 electrochemiluminescence immunoassay analyzer and its supporting reagents were used.

### Statistical Analysis

The STATA software (version 14.0, StataCorp, College Station, TX, USA) was used for statistical analysis. Continuous variables in the CRC, benign tumor, and healthy control groups were represented by the mean and standard deviation or median and interquartile range in the results. Categorized data are expressed as numbers and percentages. The frequency of lymphocyte subsets is defined as the number of cells belonging to a lymphocyte subset contained in every thousand lymphocytes. The absolute value of lymphocyte subgroup is defined as the number of million cells belonging to lymphocyte subgroup per liter of peripheral blood. According to whether the patient had hepatitis B, subgroup analysis was performed, and the differences between the two groups were analyzed using Wilcox tests. The “ggpubr” package in the R software (version 3.6.1) was used to plot the differential expression of a single variable between the CRC group, colorectal benign tumor group, and healthy control group. Significant differences were defined at *p <* 0.05.

The diagnostic performance of markers for CRC was evaluated based on the receiver operating characteristic (ROC) curve, and compared based on the size of the area under the curve (AUC). Youden Index was used to calculate sensitivity and specificity.

## Results

According to the inclusion and exclusion criteria, the study included 74 patients, comprising 33 patients with CRC and 41 patients with colorectal benign tumors. In addition, 49 healthy volunteers were simultaneously recruited as the healthy control group (**Supplemental Figure 1**). The basic characteristics of the subjects included in the study are shown in **Table 1**.

**Table 1:** Basic characteristics of the colorectal cancer, benign tumor, and healthy control groups.

Characteristics	Healthy control (N = 49)	Benign tumor (N = 41)	Colorectal cancer (N = 33)
Age (years)	63.24 ± 12.65	60.49 ± 11.79	67.09 ± 9.88
Male/Female	27 (55.10%)/22 (44.90%)	26 (63.41%)/15 (36.59%)	14 (42.42%)/19 (57.58%)
Hepatitis B	0 (0%)	6 (14.63%)	8 (24.24%)
CEA (ng/mL)	2.16 (1.58–3.12)	2.46 (1.58–3.13)	3.47 (2.08–5.98)
CA199 (U/mL)	11.85 (8.34–16.44)	11.70 (7.6–16.5)	13.1 (7.7–36.8)
Tumor site			
Colon		28 (68.29%)	15 (45.45%)
Rectum		9 (21.95%)	16 (48.48%)
Rectum & colon		4 (9.76%)	2 (6.06%)
Primary tumor (T) stage			
T1-3/T4			17 (51.52%)/16 (48.48%)
Regional lymph node (N) stage			
N0/N1-2			14 (42.42%)/19 (57.58%)
Distant metastasis (M) stage			
M0/M1			27 (81.82%)/6 (18.18%)
pTNM stage			
Stage I-II			11 (33.33%)
Stage III			16 (48.48%)
Stage IV			6 (18.18%)

### Absolute Value of CD4^+^ T Lymphocyte Subsets in CRC, Benign Tumor, and Healthy Control Groups

**Figure 2:**
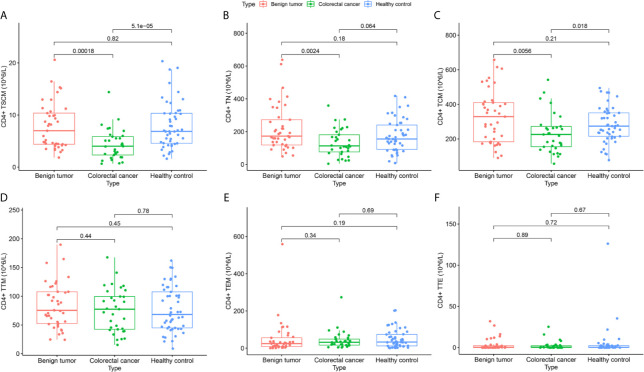
Differences in the absolute value of CD4^+^ lymphocyte subsets among subjects in the colorectal cancer, benign tumor, and healthy control groups. **(A)** CD4^+^ TSCM (10^5^/L); **(B)** CD4^+^ TN (10^5^/L); **(C)** CD4^+^ TCM (10^5^/L); **(D)** CD4^+^ TTM (10^5^/L); **(E)** CD4^+^ TEM (10^5^/L); **(F)** CD4^+^ TTE (10^5^/L).

According to the absolute value of each lymphocyte subgroup (**Table 2**), the expression level of CD4^+^ TSCM in the CRC group was 4.01 (2.32–5.86), which significantly differed from that in the benign tumor group and healthy control group (**Figure 2A**). In addition, compared with the benign tumor group, the absolute values of CD4^+^ TN [113.40 (76.66–181.41) *vs*. 172.99 (119.10–272.97), *p* = 0.0024] and CD4^+^ TCM (232.94 ± 112.03 *vs*. 328.92 ± 152.39, *p* = 0.0056) were significantly reduced in the CRC group. Compared with that in the healthy control group, the absolute value of CD4^+^ TCM (232.94 ± 112.03 *vs*. 281.67 ± 99.66, *p* = 0.018) in the CRC group was also reduced. However, the absolute values of CD4^+^ T lymphocyte subsets between the benign tumor group and healthy group were not significantly different (**Figure 2**).

**Table 2:** Expression levels of CD4^+^ T lymphocyte subsets in peripheral blood in the colorectal cancer, benign tumor, and healthy control groups.

Subset	Marker	Healthy control (N = 49)	Benign tumor (N = 41)	Colorectal cancer (N = 33)
CD4^+^ TSCM, 10^6^/L	CD4^+^CD45RO^-^CCR7^+^CD27^+^CD28^+^CD95^+^	6.86 (4.57–10.33)	6.98 (4.39–10.37)	4.01 (2.32–5.86)
CD4^+^ TN, 10^6^/L	CD4^+^CD45RO^-^CCR7^+^CD27^+^CD28^+^CD95^-^	155.33 (91.98–241.00)	172.99 (119.10–272.97)	113.40 (76.66–181.41)
CD4^+^ TCM, 10^6^/L	CD4^+^CD45RO^+^CCR7^+^CD28^+^	281.67 ± 99.66	328.92 ± 152.39	232.94 ± 112.03
CD4^+^ TTM, 10^6^/L	CD4^+^CD45RO^+^CCR7^-^CD28^+^	77.13 ± 38.88	83.62 ± 38.95	75.59 ± 37.93
CD4^+^ TEM, 10^6^/L	CD4^+^CD45RO^+^CCR7^-^CD28^-^	32.83 (11.98–73.86)	24.18 (8.81–55.64)	31.57 (16.35–48.67)
CD4^+^ TTE, 10^6^/L	CD4^+^CD45RO^-^CCR7^-^CD28^-^	0.30 (0–2.33)	0.24 (0–2.18)	0.4 (0–2.16)
CD4^+^ TSCM, ‰	CD4^+^CD45RO^-^CCR7^+^CD27^+^CD28^+^CD95^+^	4 (2.5–5.4)	3.5 (2.7–6.2)	3.1 (2.2–4.5)
CD4^+^ TN, ‰	CD4^+^CD45RO^-^CCR7^+^CD27^+^CD28^+^CD95^-^	96.17 ± 46.47	123.38 ± 68.02	101.86 ± 56.12
CD4^+^ TCM, ‰	CD4^+^CD45RO^+^CCR7^+^CD28^+^	155.69 ± 44.29	182.34 ± 61.88	178.53 ± 59.14
CD4^+^ TTM, ‰	CD4^+^CD45RO^+^CCR7^-^CD28^+^	42.75 ± 19.59	48.03 ± 19.48	59.73 ± 30.19
CD4^+^ TEM, ‰	CD4^+^CD45RO^+^CCR7^-^CD28^-^	18.8 (7.5–48.4)	15 (5.4–33.4)	23.4 (13.1–41.5)
CD4^+^ TTE, ‰	CD4^+^CD45RO^-^CCR7^-^CD28^-^	0.2 (0–1.3)	0.2 (0–1.8)	0.3 (0–1.8)

TSCM, memory stem T cell; TN, naive T cell; TCM, central memory T cell; TTM, terminal memory T cell; TEM, effector memory T cell; TTE, terminal effector T cell.

### Frequency of CD4^+^ T Lymphocyte Subsets in CRC, Benign Tumor, and Healthy Control Groups

**Figure 3:**
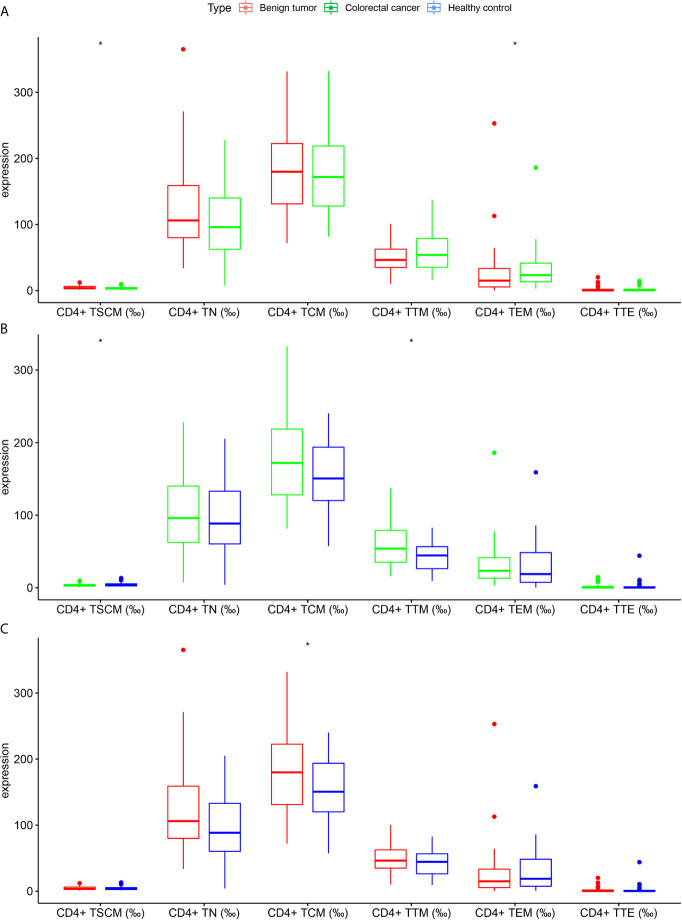
Differences in the frequency of CD4^+^ lymphocyte subsets in subjects in the colorectal cancer, benign tumor, and healthy control groups **(A)** Colorectal cancer group *vs*. benign tumor group; **(B)** Colorectal cancer group *vs*. healthy control group; **(C)** Benign tumor group *vs*. healthy control group. *p < 0.05.

As shown in **Table 2** and **Figure 3**, the frequency of CD4^+^ TSCM in the CRC group significantly differed from the benign tumor group and healthy control group (*p* < 0.05). In addition, CD4^+^ TEM significantly differed between the benign tumor group and CRC group [15 (5.4–33.4) *vs*. 23.4 (13.1–41.5), *p* < 0.05]. The frequency of CD4^+^ TTM was higher in the CRC group than in the healthy control group (59.73 ± 30.19 *vs*. 42.75 ± 19.59, *p* < 0.05). The frequency of CD4^+^ TCM between the benign tumor group and healthy control group was significant (182.34 ± 61.88 *vs*. 155.69 ± 44.29, *p* < 0.05).

### CD4^+^ TSCM in Early Screening and Auxiliary Diagnosis of Colorectal Cancer

**Figure 4:**
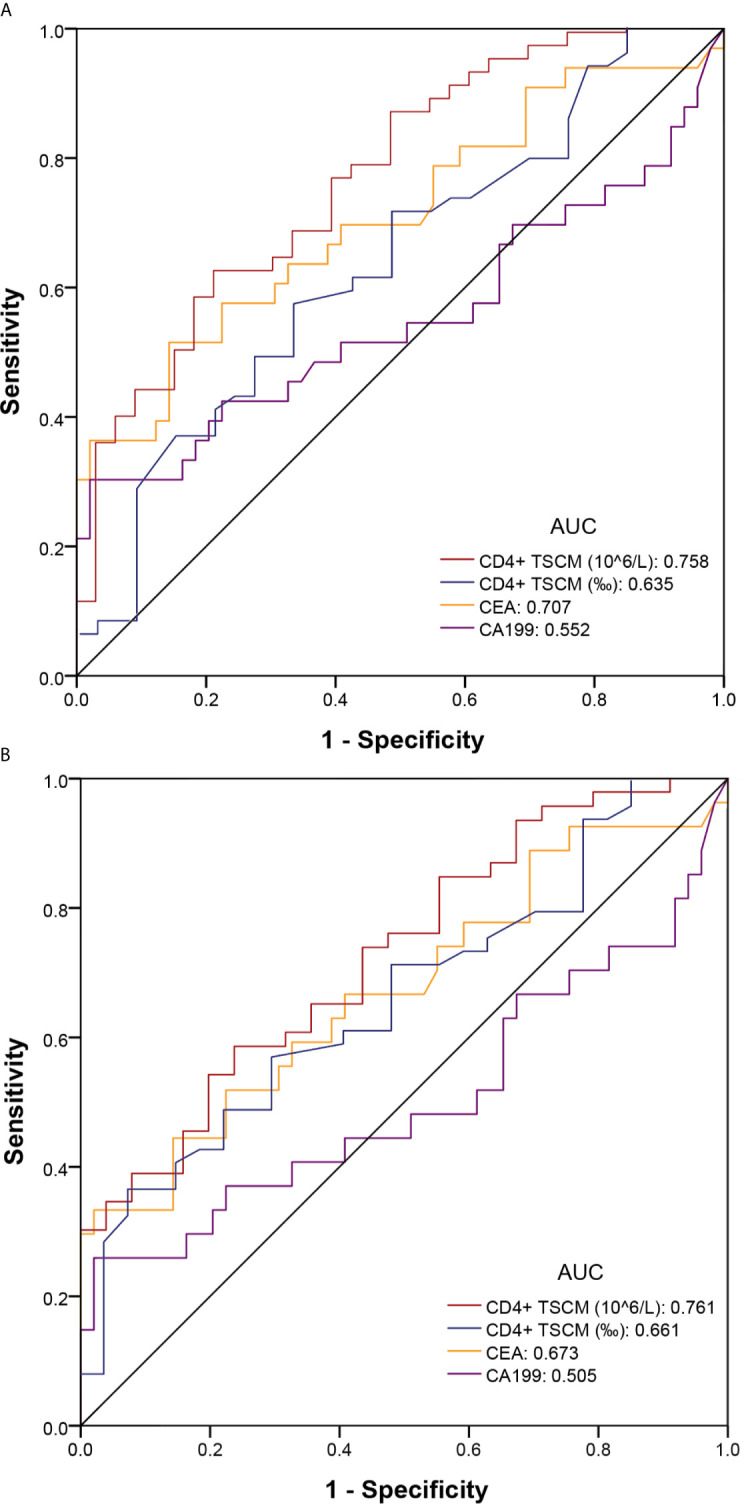
Receiver operating characteristic (ROC) curve of colorectal cancer for early screening and auxiliary diagnosis. **(A)** Colorectal cancer (pTNM stage I–IV) and healthy control groups; **(B)** Colorectal cancer (pTNM stage I–III) and healthy control groups.

The absolute value of CD4^+^ TSCM for the auxiliary diagnosis of CRC (pTNM stage I–IV), based on the AUC, was 0.758 (sensitivity: 0.612; specificity: 0.788), which was higher than that of CEA (AUC: 0.707) and CA199 (AUC: 0.552). High sensitivity is required for accurate early screening. The sensitivity of the absolute value of CD4^+^ TSCM did not decrease after removing patients with advanced CRC (pTNM stage IV) from the analysis, showing similar values as those before their removal (sensitivity: 0.612). The sensitivity of CEA and CA199 was reduced to a certain extent, and was significantly lower than the absolute value of CD4^+^ TSCM. Compared with CEA and CA199, the frequency of CD4^+^ TSCM in early screening and auxiliary diagnosis of colorectal cancer had no outstanding performance (**Table 3** and **Figure 4**).

**Table 3:** Receiver operating characteristic curve analysis to evaluate the use of CD4^+^ TSCM, CEA, and CA199 in early screening and auxiliary diagnosis of colorectal cancer.

Subset	Colorectal cancer (pTNM stage I-IV) & Healthy control				Colorectal cancer (pTNM stage I-III) & Healthy control			
	AUC	95% CI	Sensitivity	Specificity	AUC	95% CI	Sensitivity	Specificity
CD4^+^ TSCM, 10^6^/L	0.758	0.654–0.863	0.612	0.788	0.761	0.654–0.869	0.612	0.778
CD4^+^ TSCM, ‰	0.635	0.513–0.758	0.571	0.667	0.661	0.537–0.784	0.367	0.926
CEA, ng/mL	0.707	0.588–0.827	0.515	0.857	0.673	0.540–0.807	0.333	0.980
CA199, U/mL	0.552	0.413–0.691	0.303	0.980	0.505	0.353–0.656	0.259	0.980

### Subgroup Analysis Based on Patients With or Without Hepatitis B

The patients were divided into two groups according to whether they had hepatitis B infection. There were 14 patients with hepatitis B and 109 patients without hepatitis B. As shown in **Table 4**, except for the absolute value of CD4^+^ TCM, which was significantly reduced in patients with hepatitis B [177.47 (117.20–272.21) *vs*. 272.73 (204.17–363.97) (*p* = 0.025)], the frequency and absolute value of other CD4^+^ lymphocyte subsets did not significantly differ between the two groups.

**Table 4:** Differences in the frequency and absolute value of CD4+ lymphocyte subsets in patients with or without Hepatitis B.

	Patients with Hepatitis B (N = 14)	Patients without Hepatitis B (N = 109)	*P*-value
CD4^+^ TSCM, 10^6^/L	4.58 (2.57–6.00)	6.18 (4.09–9.87)	0.106
CD4^+^ TN, 10^6^/L	117.83 (70.68–186.75)	158.21 (103.99–228.23)	0.139
CD4^+^ TCM, 10^6^/L	177.47 (117.20–272.21)	272.73 (204.17–363.97)	0.025
CD4^+^ TTM, 10^6^/L	70.37 (39.32–102.38)	72.95 (47.53–107.84)	0.381
CD4^+^ TEM, 10^6^/L	18.83 (10.01–37.29)	31.28 (11.98–60.47)	0.300
CD4^+^ TTE, 10^6^/L	0.03 (0–2.06)	0.37 (0–2.33)	0.168
CD4^+^ TSCM, ‰	4.25 (1.9–7.9)	3.5 (2.5–5.1)	0.679
CD4^+^ TN, ‰	92.15 (69.4–165.7)	100.8 (62.3–136.7)	0.933
CD4^+^ TCM, ‰	173.2 (119.2–216.4)	166.1 (128–212.7)	0.930
CD4^+^ TTM, ‰	51.25 (36.4–71.5)	46.1 (30.4–63)	0.185
CD4^+^ TEM, ‰	18.65 (8.3–39)	18.8 (7.4–41.1)	0.927
CD4^+^ TTE, ‰	0.05 (0–3.2)	0.2 (0–1.5)	0.387

TSCM, memory stem T cell; TN, naive T cell; TCM, central memory T cell; TTM, terminal memory T cell; TEM, effector memory T cell; TTE, terminal effector T cell.

## Discussion

During acute immune response, T cells proliferate rapidly to ensure an effective immune response. However, during chronic viral infections and cancer, T cells are continuously stimulated by a large number of antigens. The production of T cells is less than the consumption, and eventually it will be depleted (17). Although cytotoxic (CD8^+^) T cells are a direct component of anti-tumor immunity, CD4^+^ T lymphocytes provide not only an auxiliary function of signal presentation, but also a core function by changing the tumor microenvironment (18, 19). Studies have shown that CRC cells can evade immune surveillance by damaging CD4^+^ T lymphocytes rather than CD8^+^ T lymphocytes (20). In the absence of CD4^+^ T cells, the depletion rate of cytotoxic (CD8^+^) T cells is greatly accelerated (21). In addition, some progress has been made in the use of CD4^+^ T cells, as the main body of adoptive immunotherapy, in the treatment of solid tumors (22). Therefore, in the diagnosis and treatment of malignant tumors, CD4^+^ T cells should be further examined. Nonetheless, CD4^+^ T lymphocyte subsets show heterogeneous functions (23). We analyzed the distribution characteristics of CD4^+^ T cells at various stages in patients with CRC and demonstrated the potential of using CD4^+^ TSCM as an indicator for the diagnosis and early screening of CRC (19–21, 24–26).

In previous studies, the conclusions regarding the frequency and absolute value of CD4^+^ TSCM cells in different tumor sample types were not uniform. Hong et al. (24) found that the frequency of CD4^+^ TSCM cells was higher in the lymph nodes and peripheral blood of lung cancer patients than in healthy controls, and their absolute value in lymph nodes was significantly higher than that in peripheral blood. Vahidi et al. (25) found that the frequency of CD4^+^ TSCM increased with tumor progression in the lymph nodes of breast cancer patients with lymph node metastasis. In a study of the peripheral blood of patients with gastric cancer, Wang et al. (26) concluded that the frequency of TSCM was significantly lower in gastric cancer patients than in healthy controls. This study used peripheral blood samples, which have the advantages of being countable, easy to obtain, and convenient for screening. The absolute value of CD4^+^ TSCM in the peripheral blood of patients with CRC was significantly reduced compared to that in the peripheral blood of the controls. This can be attributed to the anti-tumor effects of lymphocytes. When performing tumor immune function, T lymphocyte subsets can quickly migrate from peripheral blood to the tumor tissue (27). In addition, the frequency of peripheral blood CD4^+^ TSCM decreased significantly in patients with CRC, demonstrating that cancer can cause an imbalance in the proportion of circulating CD4^+^ T cell subsets.

CEA and CA199 in peripheral blood are widely recognized tumor markers for colorectal cancer (28). However, CEA and CA199 rarely have significantly increased levels in the early stage of cancer, making them unfavorable indicators for early colorectal cancer screening (29, 30). This study found that the absolute value of CD4+ TSCM, based on the area under the receiver operating characteristic curve, can better distinguish CRC from healthy controls, and the diagnostic sensitivity for non-advanced CRC is significantly higher than CEA and CA199 based analysis. The occurrence and development of cancer involves various stages, and systemic immune response is activated in the early stage of cancer. CD4^+^ TSCM cells, as one of the CD4^+^ T lymphocyte subsets, exhibit self-renewal ability and effector antigen specificity and are sensitive in the early stage of disease when they appear to be significantly reduced. Based on the above analysis results, we believe that the absolute value of CD4^+^ TSCM has potential application in the early screening and auxiliary diagnosis of CRC.

Chronic viral infections such as human immunodeficiency virus or hepatitis C virus infection may consume a large number of TSCM cells, complicating the analysis of CD4^+^ TSCM results. In the inclusion and exclusion criteria used in this study, patients with chronic persistent human immunodeficiency virus, hepatitis C virus, and human papilloma virus infections were excluded. Although it is not well-understood whether hepatitis B can cause CD4^+^ T cell depletion, the immune mechanism of patients with progressive damage due to chronic infection is clear (31, 32). However, as many patients have chronic hepatitis B infection, they are not suitable for direct exclusion; therefore, we conducted subgroup analysis. The frequency and number of TSCMs was affected by hepatitis B virus infection, possibly because of the relative balance between the consumption and production of CD4^+^ T cells in patients with hepatitis B infection included in this study.

To the best of our knowledge, this is the first study on CD4^+^ TSCM and colorectal cancer patients. In addition, although there are many results in this study that are not statistically significant, it does not mean that these results have no clinical significance. We believe that this article provides novel insights into the potential immune process of CRC, and also provides a reference for subsequent research. Moreover, the number of cases included in this study was small. Thus, a study with a larger sample size is necessary to verify our hypothesis.

## Conclusions

CD4^+^ TSCM in the peripheral blood may be a promising immune index for the early screening and auxiliary diagnosis of CRC. However, the impact of CD4^+^ TSCM on the peripheral blood of CRC needs to be further explored in larger sample studies.

## Data Availability Statement

The original contributions presented in the study are included in the article/**Supplementary Material**. Further inquiries can be directed to the corresponding author.

## Ethics Statement

The studies involving human participants were reviewed and approved by Ethical Review Committee of Dongyang People’s Hospital, Zhejiang, China. The patients/participants provided their written informed consent to participate in this study.

## Author Contributions

YL: writing - original draft, formal analysis, and methodology. QZ: writing - original draft, data curation. LZ: data curation, methodology, and software. All authors contributed to the article and approved the submitted version.

## Funding

This work was supported by grants from the Jinhua Science and Technology Bureau (2020-4-138).

## Conflict of Interest

The authors declare that the research was conducted in the absence of any commercial or financial relationships that could be construed as a potential conflict of interest.

## Supplementary Material

Supplementary Figure 1 Flowchart of study population screening.
