# Supplementary figures and images for: CD4+ Memory Stem T Cell in Peripheral Blood: A Promising Immune Index for Early Screening and Auxiliary Diagnosis of Colorectal Cancer
Source: Front Oncol. 2021 Jul 13;11:701738. doi: 10.3389/fonc.2021.701738 (PMC8313852; doi:10.3389/fonc.2021.701738)

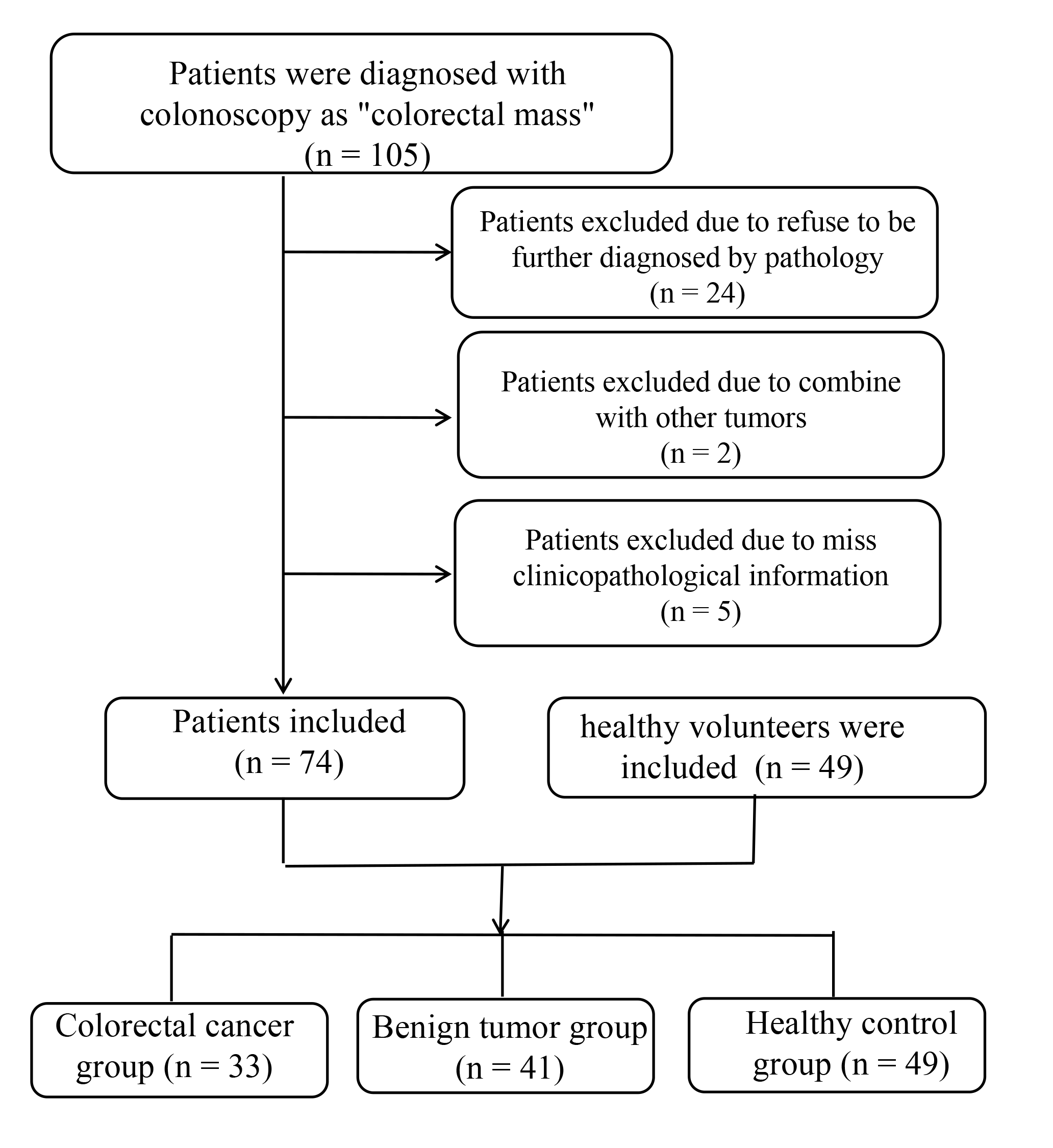

Supplement: Supplementary Figure 1 — Flowchart of study population screening. [file Image_1.tif]
